# How to start your monocot CRISPR/Cas project: plasmid design, efficiency detection, and offspring analysis

**DOI:** 10.1186/s12284-019-0354-2

**Published:** 2020-02-03

**Authors:** Jin-Jun Yue, Chwan-Yang Hong, Pengcheng Wei, Yu-Chang Tsai, Choun-Sea Lin

**Affiliations:** 1 Research Institute of Subtropical Forestry, Chinese Academy of Forestry, Hangzhou, China; 2 Department of Agricultural Chemistry, College of Bioresources and Agriculture, National Taiwan University, Taipei, Taiwan; 3 Key Laboratory of Rice Genetic Breeding of Anhui Province, Rice Research Institute, Anhui Academy of Agricultural Sciences, Hefei, China; 4 Department of Agronomy, National Taiwan University, Taipei, Taiwan; 5 Agricultural Biotechnology Research Center, Academia Sinica, Taipei, Taiwan

**Keywords:** Cas12a, Genome editing, Plant transformation, Promoter, Protoplast

## Abstract

The breakthrough CRISPR (clustered regularly interspaced short palindromic repeat)/Cas9-mediated genome-editing technology has led to great progress in monocot research; however, several factors need to be considered for the efficient implementation of this technology. To generate genome-edited crops, single guide (sg)RNA and Cas9 DNA are delivered into plant cells and expressed, and the predicted position is targeted. Analyses of successful targeted mutations have revealed that the expression levels, expression timing, and variants of both sgRNA and Cas9 need to be sophisticatedly regulated; therefore, the promoters of these genes and the target site positions are the key factors for genome-editing efficiency. Currently, various vectors and online tools are available to aid sgRNA design. Furthermore, to reduce the sequence limitation of the protospacer adjacent motif (PAM) and for other purposes, many Cas protein variants and base editors can be used in plants. Before the stable transformation of a plant, the evaluation of vectors and target sites is therefore very important. Moreover, the delivery of Cas9-sgRNA ribonucleoproteins (RNPs) is one strategy that can be used to prevent transgene issues with the expression of sgRNA and Cas proteins. RNPs can be used to efficiently generate transgene-free genome-edited crops that can reduce transgene issues related to the generation of genetically modified organisms. In this review, we introduce new techniques for genome editing and identifying marker-free genome-edited mutants in monocot crops. Four topics are covered: the design and construction of plasmids for genome editing in monocots; alternatives to SpCas9; protoplasts and CRISPR; and screening for marker-free CRISPR/Cas9-induced mutants. We have aimed to encompass a full spectrum of information for genome editing in monocot crops.

## Design and Construction of Plasmids for Genome Editing in Monocots

**Fig. 1:**
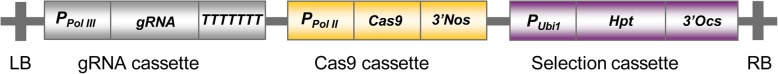
Schematic of the T-DNA region in a binary vector for genome editing in monocots. *P*_*Pol III*_: *Polymerase III* promoter. *P*_*Pol II*_: *Polymerase II* promoter. *P*_*Ubi1*_: maize *Ubiquitin 1* promoter and the first exon. *Hpt*: *Hygromycin phosphotransferase*. (Modified from Howells et al. 2018)

The CRISPR/Cas9 system has been successfully used for genome editing in a variety of monocots, including rice (*Oryza sativa*), wheat (*Triticum* sp.), barley (*Hordeum vulgare*), and maize (*Zea mays*) (Feng et al. 2018; Gasparis et al. 2018; Hu et al. 2018; Kis et al. 2019; Okamoto et al. 2017; Wang et al. 2017; Zhang et al. 2016). To perform CRISPR/Cas9-mediated genome editing, the Cas9 endonuclease is guided by a single guide RNA (sgRNA) to recognize the complementary sequence and create double-strand breaks (DSBs), thereby generating a short deletion or insertion. Genome-edited plants can be generated either by stable or transient transformation. For stable transformations, the *Agrobacterium*-mediated transformation method is typically used to deliver transfer DNA (T-DNA) into the plant cell, where it is then inserted into the plant genome (Mikami et al. 2015a; Nandy et al. 2019). For transient transformations, particle bombardment and polyethylene glycol (PEG)-mediated methods are used to deliver plasmids or ribonucleoproteins (RNPs) into the plant cells (Woo et al. 2015; Svitashev et al. 2016; Zhang et al. 2016; Lin et al. 2018). Plasmids used for the stable genome editing of plants require a selection cassette, known as a sgRNA cassette, and a clustered regularly interspaced short palindromic repeat (CRISPR)/CRISPR-associated protein 9 (Cas9) cassette in the T-DNA region, while the selection cassette is not necessary for transient transformations (Fig. 1).

1. Selection cassette: In monocots, several genes have served as useful selection markers for the efficient selection of transgenic plants, such as *neomycin*-*phosphotransferase* (*NPTII*), *bar*, mutated *acetolactate synthase* (*ALS*), plant *phosphomannose isomerase*, and *hygromycin phosphotransferase* (*Hpt*) (Miki and McHugh 2004; Hu et al. 2016). Among these genes, *Hpt* is the most widely used selection marker which confers tolerance to the herbicide hygromycin because several crops have a natural tolerance to kanamycin. Plant phosphomannose isomerase can also be used as a selectable marker for rice transformation (Hu et al. 2016). The expression of *Hpt* is usually driven by a strong constitutive promoter, such as maize *Ubiquitin 1* (*ZmUbi1*), rice *ACTIN 1*, or *Cauliflower mosaic virus* (*CaMV*) *35S*, for the ubiquitous expression of the antibiotic-tolerance gene (Mikami et al. 2015a).
2. sgRNA cassette: sgRNA is a programmable 20-nucleotide (nt) sequence that recognizes the target DNA sequence and an invariant scaffold sequence (Ran et al. 2013), then directs the Cas nuclease to cleave the *target sequence.* Two factors are important for the function of sgRNA; promoter activity and the specificity of the sgRNA. A mixed dual promoter system is generally used in CRISPR/Cas9 system. In this system, Cas9 is directed by the *RNA Polymerase II* (Pol II) promoter while sgRNA expression is regulated by a *Pol III* promoter such as *U6* or *U3*. To increase the transcription of the sgRNA, several monocot-specific *U3* or *U6* promoters have been cloned and used to direct the expression of the sgRNA, such as those from rice (Ma et al. 2015), maize (Qi et al. 2018), and wheat (Xing et al. 2014). In rice, the sgRNA driven by the *OsU6* promoter produces more transcripts than when driven by the *OsU3* promoter (Mikami et al. 2015a); however, several promoters, including *OsU3*, *OsU6a*, *OsU6b*, and *OsU6c*, have been used to direct the expression of sgRNA, and all of them could effectively direct genome editing with mutation rates of 81.4–90.0% (Ma et al. 2015; Shan et al. 2013; Xie and Yang 2013; Zhou et al. 2014). In addition to promoters, the specificity of the sgRNA for its target DNA sequence is another factor that affects the efficiency of genome editing. Several resources available on the web can be used to design highly specific sgRNAs for use with the CRISPR/Cas9 system (Table 1; modified from Zhang lab, https://zlab.bio/guide-design-resources). Nevertheless, even if these 20-nt sequences perfectly match the target gene, some sgRNAs do not work well. The online tools listed in Table 1 recommend targets with a low risk of an off-target match, but not all predicted target sequences may result in an efficient mutation. In addition, targeted DNA sequences with GC contents higher than 50% have higher genome-editing efficiencies (88.5–89.6%) than those with GC contents lower than 50% (77.2% efficiency) (Ma et al. 2015). Successive Ts in 20-nt target sequence is not good when sgRNA expression is driven by the *U3* or *U6* promoters (Wu et al. 2014).
3. Cas9 cassette: Aspects of the *Cas9* cassette that affect the mutation rate during genome editing include the expression level and codon usage of *Cas9* (Ma et al. 2015; Xie et al. 2015). Several strategies have been conducted to improve the expression of *Cas9,* including the use of a strong constitutive promoter, the addition of a translational enhancer, and the addition of nuclear localization signals. Constitutive promoters have been used to direct the expression of *Cas9*, including the *ZmUbi1* and *35S*. The Cas9 nucleases from different bacteria may have variations in the protospacer adjacent motif (PAM) sequence that they require for cleavage; therefore, many Cas9 homologs with different PAM requirements have been isolated from different bacteria. These Cas proteins are introduced in ‘Alternatives to SpCas9’ section. *Streptococcus pyogenes* Cas9 (SpCas9) is the most common Cas used in CRISPR/Cas9-mediated genome editing. It has been codon-optimized for maize (Xing et al. 2014) and rice (Miao et al. 2013) to improve its expression levels in these monocots.

**Table 1:** Web-based tools for sgRNA design

Name	Website	Reference
Benchling	https://www.benchling.com/crispr/	Benchling, CA
Broad Institute GPP	https://portals.broadinstitute.org/gpp/public/analysis-tools/sgrna-design	Doench et al. 2016
CHOPCHOP	http://chopchop.cbu.uib.no/	Labun et al. 2019
CRISPOR	http://crispor.tefor.net/	Concordet and Haeussler 2018
CRISPR-P	http://cbi.hzau.edu.cn/CRISPR2/.	Liu et al. 2017
DeskGen	https://www.deskgen.com/landing/#/	Desktop Genetics, MA
E-CRISP	http://www.e-crisp.org/E-CRISP/designcrispr.html	Heigwer et al. 2014
Horizon Discovery	https://dharmacon.horizondiscovery.com/gene-editing/crispr-cas9/crispr-design-tool/	Horizon Discovery, UK
IDT	https://sg.idtdna.com/site/order/designtool/index/CRISPR_CUSTOM	Integrated DNA Technologies, IA
Off-Spotter	https://cm.jefferson.edu/Off-Spotter/	Pliatsika and Rigoutsos 2015
Synthego	https://www.synthego.com/products/bioinformatics/crispr-design-tool	Synthego, CA
ZiFiT	http://zifit.partners.org/ZiFiT/ChoiceMenu.aspx	Sander et al. 2010

## Strategies for Multiplex Genome Editing in Monocots

One advantage of the CRISPR/Cas9 system over other crop-breeding strategies is its flexibility for multiplex genome editing (Wang et al. 2017; Wang et al. 2018). The editing of multiple functional genes allows for the rapid improvement of multiple agronomic traits at one time, while editing the *cis*-acting elements of a promoter affects transcriptional regulation. The deletion of larger fragments between two sgRNA-targeted sites on the same chromosome following the generation of multiple DSBs has been reported in many species. The CRISPR/Cas9 system has been used to delete DNA fragments ranging from dozens of bases to greater than 1 Mb (Mali et al. 2013; Shan et al. 2013). In addition, targeted deletions of 10 bp to over 200 kb between two target sites have been reported in rice (Mikami et al. 2016; Zhou et al. 2014). The elimination of the *Tos17* retrotransposon using CRISPR/Cas9 was reported in rice, providing a rapid breeding route for making reverting the agronomically important genes that have been inactivated by the insertion of transposable elements (Saika et al. 2019).

**Fig. 2:**
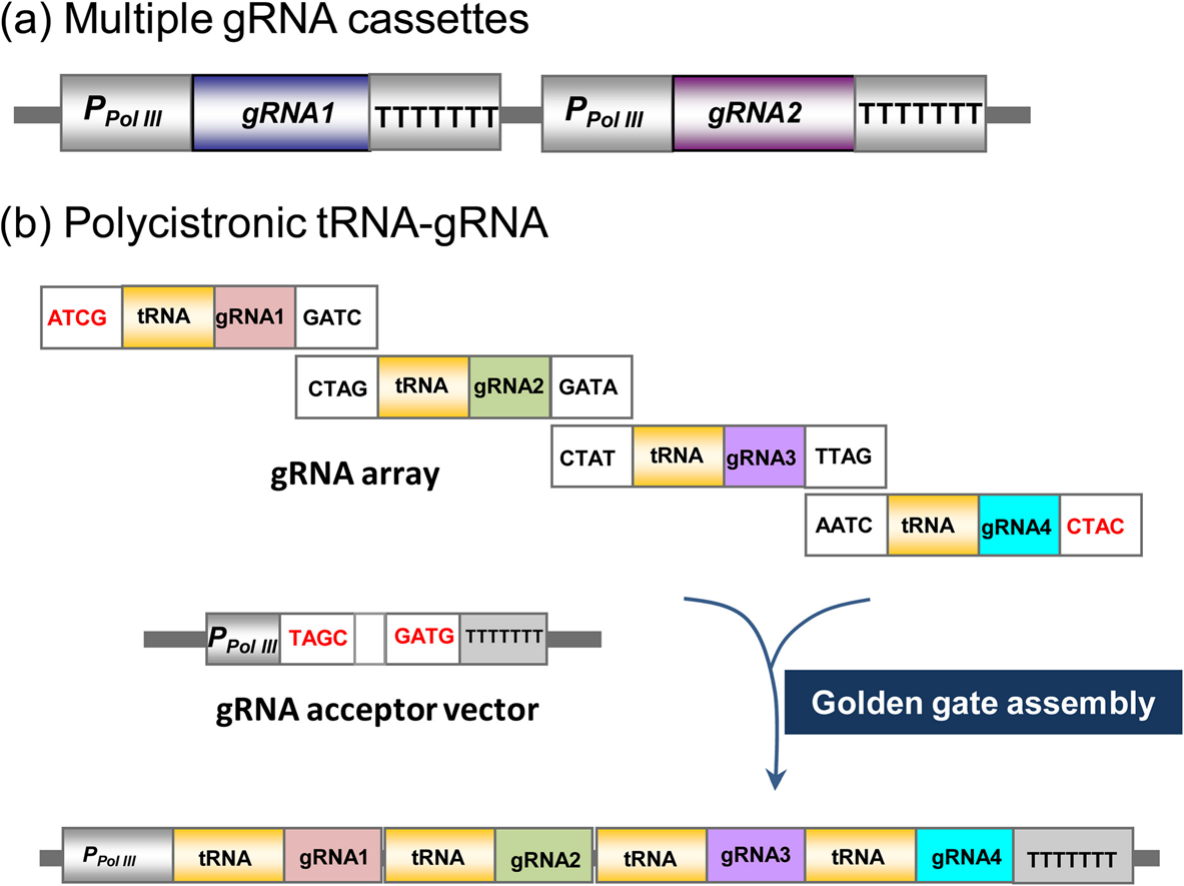
Diagram of a plasmid construct used for multiplex genome editing. **a** Stacking of multiple sgRNA expression cassettes in one T-DNA. P _*Pol III*_: *Polymerase III* promoter. **b** Cloning of four tRNA-gRNAs into the CRISPR/Cas9 binary vector using a single Golden Gate ligation. (Modified from Kurata et al. 2018)

Multiplex genome editing can be achieved by the simultaneous delivery and expression of multiple *sgRNA*s; however, since most CRISPR/Cas9 components are transferred into plants via *Agrobacterium*-mediated transformations, an efficient plasmid construction strategy is required. Traditionally, multiple *sgRNA* expression cassettes (including a *Pol III* promoter, a sgRNA, and a terminator) can be stacked into one T-DNA (Fig. 2a); however, this may increase the cloning difficulties due to the limited restriction sites available, and the fact that large T-DNAs may decrease the transformation efficiency. Alternative strategies have therefore been developed to facilitate multiplexed genome editing in plants. These alternative strategies are based on the expression of multiple *sgRNA*s as a single transcript, after which multiple functional sgRNAs are generated following the processing of the transcripts by exogenous ribozymes (Gao and Zhao 2014), Csy-type ribonuclease 4 (Csy4) (Cermak et al. 2017), or the plant endogenous transfer RNA (tRNA)-processing system (Xie et al. 2015). The endogenous tRNA-processing system exists in almost all organisms and has been successfully used to perform multiplex genome editing in rice (Xie et al. 2015). In the tRNA-processing system, the *Pol III* promoter is used to direct the expression of a single synthetic gene containing multiple tRNA-sgRNAs or a polycistronic tRNA-sgRNA (PTG) gene. The PTG gene can be generated using the Golden Gate assembly method (Lowder et al. 2015) (Fig. 2b). After transcribing the PTG, the endogenous RNases specific to tRNA would recognize the tRNA components and cleave the individual sgRNAs from the PTG transcript. The resulting sgRNAs would then guide Cas9 to multiple target sites for genome editing. Many toolboxes are currently available in the public database Addgene (Cermak et al. 2017; Castel et al. 2019; Hahn et al. 2019), from where these vectors can be purchased by academic researchers.

### Reducing off-Target Mutations and Lethality in Monocots

Several reports have indicated that constitutively expressed *Cas9* produces an excess of sgRNA-Cas9, which may increase the incidence of genome-wide off-target mutations (Hsu et al. 2013; Hu et al. 2018; Pattanayak et al. 2013; Svitashev et al. 2016). By contrast, conditionally or transiently expressing *Cas9* significantly reduces the frequency of off-target mutations (Srivastava et al. 2017; Zhang et al. 2014). Increasing evidence has indicated that conditionally expressing *Cas9* at the plant regeneration phase can markedly improve genome-editing efficiency, and this kind of conditional targeting could avoid the lethal phenotype caused by the cleavage of genes essential for development (Srivastava et al. 2017; Zhang et al. 2014). The promoter of the gene encoding heat-shock protein 17.5E (*Hsp17.5E*) from soybean (*Glycine max*) has been used to direct the expression of *Cas9* for genome editing in rice. The mutation frequency was 16% and 50–63% among the transgenic lines before and after a heat treatment, respectively (Nandy et al. 2019). In maize, *Cas9* driven by the meiosis-specific *Disrupted Meiotic cDNA 1* (*ZmDMC1)* promoter was able to generate up to 66% homozygous or bi-allelic mutants, and no off-target mutations were detected using whole-genome sequencing (Feng et al. 2018). The vector delivery of preassembled Cas9-sgRNA RNPs instead of DNA has been reported to significantly reduce the frequency of off-site cleavage in both protoplast and zygote systems (Toda et al. 2019). In maize embryo cells, the delivery of DNA vectors containing *Cas9* and sgRNA showed a high frequency of off-site mutations (50%) when compared with the Cas9-sgRNA RNP complex (0%) (Svitashev et al. 2016). In most plant species, the isolation, cultivation, and regeneration of protoplasts remains a challenge; however, recent work demonstrated the direct delivery of a Cas9-sgRNA RNP into rice zygotes using the in vitro fertilization of isolated gametes, resulting in a targeted mutation rate of 14–64%. This protoplast-free zygote system makes the RNP-mediated genome-editing system much easier to perform, which could be a potential avenue for crop improvement in many monocot species (Toda et al. 2019).

## Alternatives to SpCas9

The CRISPR/Cas systems are divided into two classes. The Class 1 systems possess multiple Cas protein subunits, whereas the Class 2 systems utilize a single, multifunctional protein effector (Shmakov et al. 2015). Class 2 CRISPR systems are further divided into types II, V, and VI. Along with the commonly used type II effector SpCas9, other orthologs with RNA-guided site-specific nuclease (SSN) activity have been engineered for use as tools in the genome editing of eukaryotic cells. These orthologs include proteins from *Staphylococcus aureus* (SaCas9), *Streptococcus thermophilus* (St1Cas9), *Francisella novicida* (FnCas9), *Neisseria meningitidis* (NmCas9), *Brevibacillus laterosporus* (BlCas9), and *Campylobacter jejuni* (CjCas9) (Cong et al. 2013; Hou et al. 2013; Sampson et al. 2013; Ran et al. 2015; Karvelis et al. 2015; Kim et al. 2017a; Chatterjee et al. 2018; Edraki et al. 2019). These Cas9s can be a welcome complement to the editing ability of SpCas9 in plants. Here, we describe plant genome-editing tools developed from these orthologs and discuss their advantages for research in botany.

### Type II Cas9 Systems

Type II CRISPR systems are abundant in prokaryotic organisms and are the principal resource used to develop gene-editing tools. In addition to the first-reported SpCas9, several studies have described the Cas9 ortholog-mediated genetic engineering of plants. SaCas9 is most frequently used in place of SpCas9 and provides comparable editing efficiency for eukaryotic genomes. Compared with SpCas9, SaCas9 may reduce the delivery barrier of the CRISPR system because of its smaller size (Friedland et al. 2015; Ran et al. 2015). SaCas9 and its engineered variant SaKKH (E782K/N968K/R1015H) (Kleinstiver et al. 2015), which relaxes the canonical NNGRRT PAM of SaCas9 to NNNRRT, have been used to achieve the efficient targeted mutagenesis of *Arabidopsis thaliana*, tobacco (*Nicotiana tabacum*), rice, and citrus (*Citrus sp.*) (Kaya et al. 2016; Steinert et al. 2015; Jia et al. 2017; Qin et al. 2019).

SaCas9 and SaKKH have also been used to develop plant base editors (BEs), including the cytosine BE (CBE) responsible for a C·G to T·A conversion and the adenine BE (ABE) responsible for the reverse substitution of A·T to G·C. Similar to the BEs derived from SpCas9, the SaCas9 BEs have been successfully used to induce specific base conversions in the rice genome (Hua et al. 2018; Hua et al. 2019; Qin et al. 2019). Notably, the editing windows of the SaCas9 BEs are much broader than those of the SpCas9 BEs, possibly due to differences in the formation of the R-loop complex (Kim et al. 2017d). Compared the conventional CRISPR/Cas9-targeted mutagenesis system, BEs developed from SpCas9 tend to be less efficient at editing certain targets and less flexible for specific nucleotides. Because of their recognition of different PAMs and the enlargement of the editing window, the SaCas9 BEs have provided alternative tools for precise genome editing in plants.

Another Cas9 ortholog, St1Cas9, has been used to induce mutations in the *Arabidopsis* gene *ALCOHOL DEHYDROGENASE 1* (*ADH1*) (Steinert et al. 2015), while FnCas9 was used to confer molecular immunity against RNA viruses in tobacco and *Arabidopsis* (Zhang et al. 2018).

### Type V Cas Systems

The CRISPR RNA (crRNA)-guided SSN activity of the type V Cas system, which includes Cas12a and Cas12b, also generates DSBs. Several Cas12a (formerly Cpf1) orthologs, such as *F. novicida* Cas12a (FnCas12a), *Acidaminococcus* sp. Cas12a (AsCas12a), and *Lachnospiraceae sp.* Cas12a (LbCas12a), have been engineered as a class of genome-editing tools distinct from the Cas9 system (Zetsche et al. 2015). The Cas12a proteins induce staggered DSBs at sites distal to a 5′ T-rich PAM, generating relatively longer deletions via the non-homologous end joining repair pathway in various plant species, including rice (Endo et al. 2016a; Begemann et al. 2017; Hu et al. 2017; Kim et al. 2017c; Tang et al. 2017; Xu et al. 2017). Similar to the SpCas9 systems, transgene-free Cas12a-mediated mutants can be generated by simply segregating the T-DNA fragment in the transition from the T_0_ to T_1_ generations (Xu et al. 2017). In addition, mixing RNP with Cas12a and crRNA has allowed transgene-free genome editing in soybean and tobacco (Kim et al. 2017b). Interestingly, the Cas12a proteins have different sensitivities to temperature in plants (Malzahn et al. 2019); therefore, the optimization of the incubation temperature used in crop culture systems may facilitate a higher editing efficiency when using Cas12a proteins.

Cas12a proteins not only induce DSBs, but also process their own pre-crRNA for maturation. This RNase activity provides a simple pattern by which single or multiplex crRNA(s) can be expressed from the same transcription unit of Cas12a, facilitating the highly efficient genome editing of plants (Wang et al. 2017; Ding et al. 2018; Wang et al. 2018; Xu et al. 2019). In the genome editing mediated by the CRISPR/Cas9 system, RNA could be used as a repair template for homology-directed repair (Butt et al. 2017). The self-processing activity of Cas12a means that homologous recombination-mediated gene replacements can be generated using the FnCas12a system (Li et al. 2019). In mammalian cells, CBEs were also developed using Cas12a proteins to facilitate precise C-to-T conversions in A/T-rich regions (Li et al. 2018); however, similar systems have not yet been reported in plants. The engineered *Alicyclobacillus acidiphilus* Cas12b (AaCas12b) has been used to edit mammalian genomes under a wide range of temperatures (31–59 °C; Teng et al. 2018), but it is still unclear whether the high-temperature-preferring Cas12b systems function in plants.

### Type VI Cas Systems

The type VI CRISPR proteins, such as Cas13a, can target and cleave the target RNA under the guidance of crRNA. The heterologous expression of *Leptotrichia wadei* Cas13a (LwaCas13a) causes the knockdown of target RNAs in mammalian and plant cells (Abudayyeh et al. 2017). Similarly, in both dicot and monocot plants, the transient or stable expression of a Cas13a-crRNA complex results in the targeting and degradation of the RNA transcripts of endogenous genes and foreign reporters (Aman et al. 2018; Zhang et al. 2019a, 2019b). To precisely edit nucleotides in RNA, the ADAR2 adenosine deaminase or an evolved ADAR2 cytidine deaminase was fused to a catalytically inactive Cas13a (dCas13a), generating C-to-U and A-to-I RNA editors in mammalian cells (Cox et al. 2017; Abudayyeh et al. 2019). RNA editing, especially organellar RNA editing, plays an irreplaceable role in plant growth and development; therefore, similar Cas13a tools are highly anticipated to facilitate related research in plants.

## Protoplasts and CRISPR

### Protoplast Isolation and Validation

Many different vectors are available for the expression of Cas proteins using different promoters. Researchers should choose a suitable vector based on their needs, and importantly should evaluate the target sites and constructs using a transient expression system before performing the stable transformation to reduce the time and labor required. Protoplasts are often used for plant science investigations (Marx 2016), and the convenience and speed of their transfection means they are an attractive model in which to assess the mutagenesis efficiency of a CRISPR/Cas system, including the validation of Cas protein codon optimizations or modifications, sgRNA target sites, the promoters used for sgRNA and Cas9 proteins, and different vector designs (Andersson et al. 2017; Butt et al. 2017; Cermak et al. 2017; Endo et al. 2019; Hsu et al. 2019; Hsu et al. in preparation; Li et al. 2013; Li et al. 2018; Liang et al. 2014; Lowder et al. 2015; Shan et al. 2013; Sun et al. 2015; Zong et al. 2017, 2018). Rice, tobacco, and soybean protoplasts have been used to analyze Cas9 and Cas12a (Kim et al. 2017; Tang et al. 2017), while Cas13a was examined in rice protoplasts (Abudayyeh et al. 2017). Protoplast transfection can also be used to evaluate the efficiency of the use of RNPs (Andersson et al. 2018; Hsu et al. in preparation; Kim et al. 2017; Malnoy et al. 2016; Woo et al. 2015).

In comparison with the number of articles on stable rice CRISPR transformations, very few have been published using protoplasts for validation. One of the reasons for this is the efficiency of protoplast transfection and isolation. We have evaluated the different methods of protoplast transfection in rice, including PEG, electroporation, and liposome delivery, which revealed that the PEG method has highest transfection efficiency (Lin and Hsu, personal comm.). The main bottleneck restricting the application of rice protoplasts in the evaluation of genome-editing reagents is thus considered to be protoplast isolation. We previously improved the protocol for isolating *Arabidopsis* protoplasts (Tape *Arabidopsis* Sandwich; Wu et al. 2009) to facilitate their use for various purposes, and we are currently trying to develop a convenient rice protoplast isolation method, modifying two of the steps reported in previous rice mesophyll protoplast isolation protocols (Chen et al. 2006; Zhang et al. 2011).

First, based on our observations, seedlings cut cross-sectionally retain more cells within the leaf sheath after digestion, meaning the mesophyll cells can be digested but not released. The veins in rice run parallel to each other; therefore, we changed the cut direction from a random or cross-sectional cut to a longitudinal cut parallel to the veins, which allows the enzyme solution to more easily access the cells and provides more surface area from which the protoplasts are released. To increase the efficiency of this process, multiple blades were fixed by a holder, creating a tool that can increase the speed of cutting (Lin et al. 2018). Second, we also assessed the enzyme components required for the digestion solution when using longitudinal cuts, revealing that the less expensive Cellulase R10 (Yakult, Japan) could be used in place of the more expensive Cellulase RS (Yakult, Japan) solution typically used for rice (Zhang et al. 2011). This protocol can also be applied to other Poaceae species, including wheat, bamboo (*Bambusa oldhamii*), millet (*Setaria italica*), and maize (Lin et al. 2018). We believe this convenient method will be of benefit not only in rice, but also for Poaceae crop research in general. We have also established protoplast isolation protocols for use with the Solanaceae and Cruciferae (Hsu et al. submitted). The protoplasts isolated using these methods can be used to more rapidly evaluate the genome-editing efficiency in crops (Lin et al. 2018).

### Single Protoplast Analysis

When using protoplasts to validate CRISPR efficiency, more than 100,000 protoplasts are typically used in each transfection experiment. DNA is extracted from the pooled protoplasts to enable the amplification of the target region using PCR. The pooled protoplasts also contain unedited DNA, making mutations difficult to detect if the mutagenesis efficiency is low (Lin et al. 2018). Mutagenesis efficiency can also be assessed using next-generation sequencing, from which the density ratio of the target fragments or the editing percentage can be determined. This method is relatively accurate, but it is expensive and time consuming. Recently, a convenient and reliable protocol for evaluating CRISPR mutagenesis efficiency from a single cell was established (Lin et al. 2018). In this approach, single cells can be isolated from various species and subjected to two rounds of PCR amplification and enzyme digestion without DNA purification to identify successful mutants. The mutated sequences and the mutation efficiency could thus be analyzed directly, allowing even low-efficiency mutation events to be detected in maize. This single-cell analysis technique could be used to improve the precision and application range of CRISPR gene editing using protoplasts.

Although these convenient methods could be used for mesophyll isolation to provide the materials for the evaluation of CRISPR editing efficiency and accuracy, it is important to consider the correlation between CRISPR efficiencies using mesophyll protoplasts and stable transformation, particularly in rice, for which a callus is typically used as the material for stable transformation (Kaya et al. 2016). Certain target sites were found to have a high CRISPR efficiency in stable transformation experiments, but their use did not result in mutations in the mesophyll protoplasts (Toki, Endo, and Lin, personal comm.). We are therefore working on developing a rice protoplast isolation protocol using callus materials, which will enable the assessment of the gene-editing relationship between these protoplasts and the stably transformed calli.

### CRISPR-Edited Protoplast Regeneration

In addition to the validation of transformation efficiency, mutated protoplasts have the advantage of being able to regenerate into entire mutant plants. Protoplasts isolated from meristematic tissues or totipotent cells were first used for plant regeneration in the early 1970s (Takebe et al. 1971), and just a few years later, researchers used protoplasts as materials for plant transformation (Marton et al. 1979). The progeny of *N. tabacum* regenerated from transformed protoplasts displayed the mutant phenotype, indicating that the transgenic tumor markers (octopine and nopaline) were inherited through meiosis (Wullems et al. 1981a; Wullems et al. 1981b). Monocot protoplast regeneration (Abdullah et al. 1986; Fujimura et al. 1985; Rhodes et al. 1988a) and transformation (Rhodes et al. 1988b; Shimamoto et al. 1989; Toriyama et al. 1988) protocols have also been established.

Cas proteins and sgRNAs are sufficient for CRISPR/Cas genome editing and are no longer required once the genes have been edited. Transient expression or the direct delivery of sgRNAs and Cas proteins into the cells is sufficient for editing; therefore, the DNA encoding the Cas proteins and sgRNAs does not need to be integrated into the genome for their continued expression, making the plants regenerated from these edited cells transgene-free. In 2015, Prof. Jin-Soo Kim’s group published a milestone article using protoplasts (Woo et al. 2015), in which RNP was used as the genome-editing reagent to edit lettuce (*Lactuca sativa*) protoplasts, which were subsequently regenerated into transgene-free plants.

There are several advantages to generating transgene-free edited crops using protoplasts:

1. Protoplast transformation can be applied to edit hybrid and long-juvenile-phase crops, which are typically propagated using vegetative methods. Traditionally, the use of *Agrobacterium tumefaciens*-mediated or other stable transformation techniques means the transgene (selection markers, sgRNA, and Cas9) must be integrated into the genome. In inbred crops, such as rice, this transgene can be removed through crossing; however, this causes the segregation of the desired traits in the offspring. This separation issue also occurs in systems with a low editing efficiency, in which the transformants must be crossed in order to obtain the homozygous genotype. By contrast, homozygous edited crops can be achieved from heterozygous edited protoplasts using a second transfection (Hsu et al. 2019). Several methods are available for achieving this goal, including the delivery of RNP into the callus using a biolistic approach (Liang et al. 2017, 2019) or the transient expression of *Cas9* and *sgRNA* using an *Agrobacterium*-mediated transfection (Chen et al. 2018); however, the gene-editing/mutation efficiencies of these methods are low (less than 10%). The protoplast transformation strategy had a higher efficiency; for example, over 50% of tobacco protoplasts were mutagenized using this technique, which was similar to the numbers transformed during the efficiency evaluation without antibiotic selection.
2. Protoplast transformation can deliver more than one reagent. The current *Agrobacterium* vectors used for stable transformations are multiplex sgRNAs. The genes encoding Cas proteins are large, and no multiplex Cas protein vectors are currently available. Three different Cas protein vectors (SaCas9, FnCas12a, and nCas9-Target-AID) can be delivered into a single protoplast to edit three different target sites without interference (Hsu et al. 2019). The introduction of these three Cas protein vectors requires three subsequent *Agrobacterium-*mediated transformations, the crossing of individual mutants, or the co-transformation of *Agrobacterium* harboring independent Cas9s and different selection markers.. In addition to sgRNA and Cas proteins, donor DNA fragments are also required for knock-in genome editing, and must be delivered using *Agrobacterium* (Endo et al. 2016b; Miki et al. 2018;, Wolter and Puchta 2019). Using these protocols, only a few copies of donor DNA can be used in genome editing, which may be one of the reasons for the low efficiency of knock-in genome editing. Although multiple T-DNAs can be delivered into plant nuclei to increase the editing efficiency, this also increases the difficulty of removing the T-DNAs to produce marker-free plants. In protoplasts, however, the donor DNA can be delivered in microgram quantities, which may help increase the knock-in efficiency. Not only can the sgRNAs, Cas proteins, and donor DNAs be more easily delivered into protoplasts, but the application time and amounts of genome-editing reagents can be controlled to improve the editing efficiency.
3. Using RNPs, the issues of promoter and codon modification in different species can be solved. The original Cas proteins were obtained from microorganisms; therefore, their codons must be modified for vector construction in plant species, and their expression must be driven by plant promoters. These issues have been investigated in *Arabidopsis*, resulting in the identification of a T-DNA architecture causing homozygous mutations in the first generation after transformation (Castel et al. 2019); however, the transcription and translation of these genes can still be problematic in different target crops. The resulting amount of Cas proteins in the cell can cause a low editing efficiency; however, in protoplasts, RNPs can be used to solve this problem and provide a higher delivery efficiency for transfection.
4. With the exception of *Arabidopsis*, most *Agrobacterium*-mediated transformation protocols were performed using a tissue culture platform. In dicots, the regenerated plants were derived using organogenesis, meaning they were derived from multiple cells. Many edited transformants are therefore chimeric (Kaya et al2016). To validate their edited sequences, the transformant DNAs were sequenced. Diploids contain two alleles in each cell; therefore, if the transformants contained more than two alleles they were determined to be chimeric (Kaya et al. 2016). If the edited alleles are not present in the reproductive organs, the alleles cannot be passed on to the progeny. In contrast, protoplasts are single cells that are edited before the first cell division is completed. The regenerates are then derived from a single edited protoplast, meaning all cells have same genomic background, which enables the edited alleles to be transmitted to the next generation. In our previous studies, non-chimeric regenerates were derived from protoplasts edited using the Cas proteins Cas9, Cas12a, and Target-AID, and the genotypes were inherited in a Mendelian manner (Hsu et al. 2019). This phenomenon was also reported in lettuce (Woo et al. 2015).

RNPs (Andersson et al. 2018; Woo et al. 2015) and plasmids (Andersson et al. 2017; Zong et al. 2018) have been delivered into lettuce and potato (*Solanum tuberosum*) protoplasts, which were subsequently regenerated into transgene-free genome-edited plants. In our lab, we established *N. tabacum* (Hsu et al. 2019; Lin et al. 2018), rapid cycle brassica (*Brassica oleracea*), wild tomato (*Solanum peruvianum*), and *N. benthamiana* (Lin et al. in preparation) protoplast regenerations, and used these systems to establish RNP and plasmid DNA gene-editing platforms (Hsu et al. in preparation). Some issues are yet to be resolved in protoplast regeneration during genome editing, however:

1. The regeneration protocol is difficult to establish. Only a few protocols for genome editing and protoplast regeneration have been developed, all of which were achieved in dicots (Andersson et al. 2017, 2018; Hsu et al. 2019; Jin et al. 2019; Lin et al. 2018; Tuncel et al. 2019; Woo et al. 2015). Many protoplast regeneration protocols are available in other species, including rice (Shimamoto et al. 1989; Toriyama et al. 1988) and other important Poaceae species (Rhodes et al. 1988a, 1988b). Recent discoveries have elucidated the mechanisms by which plants can regenerate, which could be applied to improve protoplast regeneration in the future (Lowe et al. 2016). In rice, it is possible to solve the lack of an efficient protoplast regeneration protocol using an alternative method; Cas9-sgRNA RNPs could be directly delivered into rice zygotes, which can then be cultured into mature plants (Toda et al. 2019). A total of 14–64% of the transgene-free plants obtained using this method were found to contain the target mutations.
2. Unexpected mutations can occur during protoplast regeneration. When 15 protoplast potato regenerants were sequenced, they were found to include a variety of mutations, including insertions/deletions, chromosome rearrangements, and aneuploidy (Fossi et al. 2019). Indeed, somaclonal mutations occur in all tissue culture strategies, including micropropagation and somatic embryogenesis. In our experience using bamboo, somaclonal mutations occurred after a long-term subculture (Lin and Chang 1998; Lin et al. 2006, 2007; Liu et al. 2007). Using a shorter period for the protoplast regeneration or reducing the amount of supplemental plant growth regulators provided may reduce this mutation rate (Lin and Hsu, personal comm.). CRISPR/Cas can also introduce off-target mutations, with the mutation rate dependent on the CRISPR/Cas system; for example, in rice, CBE but not ABE induces genome-wide off-target mutations (Jin et al. 2019). Fortunately, off-target mutations do not have a major impact on crop breeding like they do in medical applications (Tang et al. 2019); rather, they simply result in more than one edited transformant for each transformation. In addition, mutation breeding is a strategy used in traditional breeding. We can select the transformants with good traits and use these edited lines as parental lines during crop production. In our opinion, although mutations can occur during regeneration, the protoplast regeneration techniques currently available are useful tools for transgene-free genome editing.

## Screening for Marker-Free CRISPR/Cas9-Induced Mutants

Genome editing is widely used produce new genetic variants in plants. Several approaches for creating genome-edited crops have been developed, including the CRISPR/Cas system, which can be used with various tissue types including protoplasts (as described in the previous section), callus, leaf discs, and germline cells. *Agrobacterium-*mediated, PEG-mediated, particle bombardment, and virus infection transformation methods are commonly used for the delivery of the CRISPR/Cas system into plants, resulting in stable or transient expression patterns.

The transient expression of the CRISPR/Cas system can deliver DNA-based *sgRNA* and *Cas9* RNP sequences or the proteins themselves (Chen et al. 2018; Zhang et al. 2016). In a DNA-based CRISPR/Cas9 system, recombinant DNA can be transferred to the plant using *Agrobacterium-* or PEG-mediated transformations or particle bombardment, eliminating the need for herbicide or antibiotic selection steps. This method allows the expression of the CRISPR/Cas9 plasmid without requiring its integration into the plant genome. This approach can reduce regeneration time via tissue culture while producing mutation frequencies similar to the stable expression of the CRISPR/Cas9 vector at the target site. More than 86% of wheat T_0_ mutants generated using this technique were transgene free (Zhang et al. 2016). In the Transgene-free CRISPR/Cas9 system, RNPs are assembled in vitro and directly delivered into the protoplasts using a PEG fusion approach. The RNP complex directly targets the recognized sequences and induces DSBs (Park and Choe 2019; Woo et al. 2015). The binding of the RNP complex to the target DNA is tight, and the half-life for dissociation is slow (more than 6 hours in vitro) (Didovyk et al. 2016). After dissociation, the RNP complex is degraded quickly in the cell.

The majority of genome-edited plants involve the stable integration of the CRISPR/Cas9 system into the plant genome. CRISPR/Cas9 DNA is delivered by methods similar to those employed for transient expression, followed by a herbicide or antibiotic selection of successful transformants containing a marker gene. Antibiotic and herbicide resistance markers have been widely used in plant biotechnology (Wilmink and Dons 1993); however, genome-integrated CRISPR/Cas9 carries a risk of increased off-target effects and requires researchers to follow the current regulations for typical genetically modified crops. To bypass these strict biosafety regulations, CRISPR/Cas binary vectors containing selection markers or foreign DNA can be segregated in the progeny by self-pollinating or crossing the transformants (Gao et al. 2016). The PCR amplification of vector sequences can be used to verify the presence of foreign DNA in the genome. A rapid method using antibiotics to identify marker-free genome-edited plants was also reported recently (Wu et al. 2019). Leaf sections from the T_1_ progeny of genome-edited rice were incubated with hygromycin B, an antibiotic commonly used for the positive selection of transgenic plants. In the presence of hygromycin B, the genome-edited rice plants that did not retain CRISPR/Cas vectors in their genome could produce reactive oxygen species (e.g., H_2_O_2_) in their mitochondria and chloroplasts (Oung et al. 2015). These elevated H_2_O_2_ levels can be visualized directly using 3,3’diaminobenzidine (DAB) staining (Wu et al. 2019). This approach can be easily applied to most monocot species.

### Identification of CRISPR/Cas9-Induced Mutations

Following the delivery of the CRISPR/Cas system into monocot calli or other tissues, a T_0_ generation of plants harboring edited genes is regenerated. Three categories of site-directed nuclease systems (SDN1–3) are employed in genome-editing techniques (Podevin et al. 2013). SDN1 relies on the most common endogenous processes of non-homologous end-joining to repair DSBs in the plant DNA. This process is error prone, and may result in random mutations at the break site (Bortesi and Fischer 2015). SDN2 involves homology-directed repair using one or a few nucleotides as a template (EFSA Panel on Genetically Modified Organisms 2012). SDN3 uses the same repair mechanism as SDN2 but with a longer nucleotide template. Unlike SDN1, the repair processes used by SDN2 and SDN3 is not random, and does not cause substitutions, insertions, or deletions at the repair sites.

Researchers have developed several methods to increase the efficiency of screening large numbers of mutants (Table 2). These methods can detect on-target or off-target variants and include the restriction enzyme (RE) cleaved amplified polymorphic sequences (CAPS) assay (Shan et al. 2014), RE site created assays (Hodgens et al. 2017), T7 endonuclease I assays (T7E1) (Vouillot et al. 2015), polyacrylamide gel electrophoresis (PAGE)-based genotyping assay (Zhu et al. 2014), high-resolution melting analysis (HRM) (Thomas et al. 2014), PCR- and labeling-based method (Biswas et al. 2019), and annealing at critical temperature PCR (ACT-PCR) (Hua et al. 2017).

**Table 2:** Advantages and disadvantages of different methods for CRISPR/Cas9-mediated mutant screening. (Adapted from Bao et al. 2019)

Method	Advantages	Disadvantages	Reference
CAPS A RE site within the DNA target site is destroyed by a genome-editing mutation	Simple, fast, economical, and can detect homozygous and heterozygous mutants	Limited to the original target sequences	(Shan et al. 2014)
indCAPS A RE site is created using mismatch primers next to a DNA target site	More flexibility for different types of indel	Requires designing specific primers to distinguish known indel alleles	(Hodgens et al. 2017)
T7E1 cleavage assay T7 endonuclease 1 digests mismatched heteroduplexes formed between wild-type strands and mutated strands	Simple, fast, economical, and can detect heterozygous mutants	Cannot detect homozygous mutants	(Vouillot et al. 2015)
PAGE Homoduplex DNA migrates faster than heteroduplex DNA in native PAGE	Simple, fast, economical, and can detect homozygous and heterozygous mutants	Time consuming and low throughput	(Zhu et al. 2014)
HRM Homozygous DNA has a unique melting temperature (Tm), while mutated heterozygous DNA has a lower Tm	Fast and efficient for detecting SNPs and indels in mutants	Requires specific instrumentation and sensitivity is affected by amplicon size	(Thomas et al. 2014)
ACT-PCR A critical annealing temperature in PCR suppresses the mismatched annealing of the primer to the template, inhibiting the production of amplicons	Simple, fast, economical, and can detect homozygous mutants	Requires designing specific primers and is time consuming and/or labor intensive	(Hua et al. 2017)
PCR- and labeling-based assay	Simple, effective, and sensitive	Not able to reveal the exact nucleotide change in the mutant	(Biswas et al. 2019)
Whole-genome sequencing	Identifies on-target and off-target mutations	Costly and time consuming	(Tang et al. 2018)

The CAPS, indCAPS, and T7E1 cleavage assays for identifying gene-edited mutants are based on an enzymatic approach. Wild-type and mutant sequences are amplified using PCR then subjected to enzyme digestion. A typical CAPS assay can be used if a RE cutting site is present at the CRISPR target site, which is disrupted once the sequences are mutated; however, RE sites are not always present within target regions. A similar dCAPS assay has been developed for genome regions not possessing different RE sites between the wild type and the mutant. This dCAPS assay introduces or disrupts a RE site near the mutation site by amplifying several mismatched nucleotides, which greatly increases the flexibility for selecting the sgRNA target sites. T7 endonuclease 1 can be useful for digesting the mismatched heteroduplexes formed between the wild-type and mutated strands; however, this method lacks the sensitivity to distinguish between homozygous mutants and the wild type.

Other PCR-based analyses include PAGE, HRM, ACT-PCR, and PCR−/labeling-based assays. To distinguish genome-edited mutations from wild-type target sequences, target region amplicons are migrated on a native polyacrylamide gel. Homoduplex DNA migrates faster than heteroduplex DNA. To distinguish homozygous mutants from the wild type, unknown amplicons can be pre-mixed with wild-type amplicons before the assay. The migration pattern of the pre-mixed homozygous mutant and wild type will be similar to that of the heterozygous mutant. HRM is a fluorescence-based technique for determining the differences in the melting temperatures of heteroduplex and homoduplex DNA fragments. This technique can detect differences as small as 0.1 °C; however, its sensitivity is influenced by the amplicon length and variation of the mutated sequence. ACT-PCR assay can also distinguish homozygous mutant sequences at the target site using optimal annealing temperature and specific primers; however, the assay requires the design of specific primers and is time consuming and labor intensive. A simple PCR- and amplicon labeling-based method was recently used to identify the CRISPR/Cas9-generated mutants in rice (Biswas et al. 2019). This approach requires two pairs of primers or a FAM-labeled allele-specific primer. The sensitivity, precision, and reliability of the FAM-labeled method allows for the detection of indels with a high sensitivity (down to ±1 bp).

Although all these PCR-based analyses enable the effective, accurate, and economical screening of CRISPR/Cas9-generated mutants, the identification of sequence changes resulting from SDN1-generated mutations requires the Sanger sequencing of amplicons generated from the target region. In addition, whole-genome sequencing is a powerful tool for identifying not only on-target and off-target mutations, but also transgene-free plants produced by genome editing. The only drawback of whole-genome sequencing is its cost and time requirement.

## Data Availability

Not applicable.
